# Functional and genomic evaluation of novel exopolysaccharide produced by marine *Pediococcus pentosaceus* E3 with antidiabetic, anticancer, and anti-inflammatory potentials

**DOI:** 10.1186/s12866-025-04370-0

**Published:** 2025-10-03

**Authors:** Nancy M. El Halfawy, Eman H. Zaghloul

**Affiliations:** 1 Botany and Microbiology Department, Faculty of Science, Alexandria University, Alexandria, Egypt; 2 National Institute of Oceanography and Fisheries (NIOF), Cairo, Egypt

**Keywords:** *Pediococcus pentosaceus*, Lactic acid bacteria, Exopolysaccharide (EPS), *eps* gene cluster, Anti-inflammatory

## Abstract

Lactic acid bacteria (LAB) exopolysaccharides (EPS) are highly valuable due to their unique structure and functional properties. *Pediococcus pentosaceus* E3 is a promising marine probiotic strain. An investigation of the E3 genome identified a gene cluster responsible for EPS production, comprising 13 genes organized into four regions: the regulatory region for EPS expression, the chain length determination region, genes that catalyze the biosynthesis of EPS repeat units, and genes for polymerization and EPS transportation. Furthermore, a total of 16 key enzymes involved in the nucleotide sugar biosynthesis pathway were predicted according to the KEGG metabolic pathways in the E3 genome sequence. Therefore, the current study investigates the characteristics and bioactivities of E3-EPS. E3 strain was grown in MRS broth supplied with 1.0% sucrose for EPS production, and E3 produced a significant quantity of EPS (400 mg/L). Structural characteristics of E3-EPS were investigated through carbohydrate content determination, FTIR, SEM, EDX, TGA, HPLC, and NMR. HPLC analysis revealed that E3-EPS is a heteropolysaccharide composed of four sugar moieties: galactose, glucose, mannose, and fucose. Moreover, E3-EPS demonstrated promising bioactivities, as its anticancer activity was evaluated against colon cancer cell lines, and the IC_50_ value was determined to be 77.05 ± 0.24 µg/mL. E3-EPS inhibited α-amylase activity by 58.3% and 82.8% at 10 and 100 µg/mL concentrations, respectively. Additionally, E3-EPS successfully decreases the expression levels of inflammatory cytokines (TNF-α and IL-6). The findings of this study suggest that the safe marine probiotic *P. pentosaceus* E3 is a source of unique EPS suitable for pharmacological applications.

## Introduction

Exopolysaccharides (EPS) are high molecular-weight extracellular carbohydrate biopolymers produced by microorganisms, including lactic acid bacteria (LAB) [28]. EPS is produced as a slimy layer that can be discharged into the surrounding environment or adhered to the cell's surface to form a capsule [48]. The production of EPS significantly influences the physicochemical properties of the cell surface, thereby conferring enhanced resilience against dehydration, detrimental environmental factors, phagocytic engulfment, and phage infection [28, 32]. In addition, EPS polymers play a significant role in biofilm formation, facilitating cell adhesion and influencing strain-specific host interactions [7, 24]. The diversity of the EPS biopolymer is due to the variations in the sugar building blocks, anomeric configuration, glycosidic linkage, and molecular weight [50]. Otherwise, the unique physicochemical properties of the EPS contribute to a spectrum of potential applications [23]. For instance, EPS produced by LAB is associated with numerous functional foods, therapeutic agents, and health benefits [23]. Thus, the potential EPS production by a safe probiotic strain is necessary to avoid the pathogenicity of the producing strains during large-scale processes [51]. Moreover, the underexplored marine environment provides a viable setting for novel LAB strains that can produce novel EPS with distinctive structures and varied biological activities, as they thrive in a harsh and challenging habitat subjected to extreme conditions [49].

The diversity of glycosyltransferases found in the gene clusters responsible for exopolysaccharide (EPS) biosynthesis is indicative of the various structures of EPS. Additionally, bacterial genomes may contain multiple gene clusters that encode for polysaccharide biosynthesis [50]. These genes play essential roles in regulation, chain-length determination, repeat-unit assembly, polymerization, and the export of EPS [3]. The primary mechanisms for bacterial polysaccharide production include Wzx/Wzy-dependent pathways, ATP-binding cassette transporter-dependent pathways, synthase-dependent pathways, and extracellular synthesis pathways [40].

Inflammation is an innate defensive reaction to illness, disorder, injury, and stress that negatively impacts the host’s immune system. Cytokine storm is a potentially fatal condition characterized by the uncontrolled and increased release of cytokines that promote inflammation, such as interleukin IL-1β, IL-6, tumor necrosis factor-alpha (TNF-α), etc. It is associated with various diseases that might lead to death, including chronic hepatitis, rheumatoid arthritis, colon cancer, atherosclerosis, multiple sclerosis, and inflammatory bowel disease (IBD) [52]. IBD, such as ulcerative colitis or Crohn's disease, are long-lasting inflammatory disorders that affect the gastrointestinal system. IBD is believed to result from multiple factors, such as an unhealthy diet and immunological reactions. IBD is characterized by symptoms such as abdominal pain, weight loss, and bloody stool, as well as inflammatory responses in the intestinal mucosa, which involve an influx of neutrophils and macrophages that release cytokines [17, 26].

Prior studies have indicated a strong association between the development of IBD and cytokines that promote inflammation, including IL-1β, IL-6, and TNF-α. Thus, the use of antibodies that counteract pro-inflammatory cytokines has been explored as a potential treatment for IBD. Multiple studies have proposed different biological variables (such as anti-TNF-α, anti-integrins, or anti-ILs) and small molecules (such as tofacitinib, a non-selective Janus kinase (JAK) inhibitor) as potential candidates for treating IBD. Nevertheless, because of the safety concerns associated with these products, such as headaches, dizziness, dermatologic effects, and acute allergy, additional research is necessary to explore the creation of medications and nutritious functional foods for IBD [26].

Recently, the rising significance of inflammation in the pathogenesis of both type 1 and type 2 diabetes, along with related metabolic illnesses, has sparked growing interest in targeting inflammation to enhance disease prevention and management. Following a comprehensive examination of the processes influencing the metabolic patterns in Type 1 and Type 2 Diabetes, as well as the associated inflammatory pathways, it is more evident that future research should prioritize a model of concurrent suppression for several inflammatory response pathways [37, 43].

*Pediococcus pentosaceus* E3 is a safe, fully sequenced marine lactic acid bacterium (LAB) with promising probiotic potential. It can produce metabolites, including lactic acid, EPS, and bacteriocin, that confer utility in preserving food and pharmaceutical potentials [47]. Therefore, this study focuses on investigating the genome of the marine *P. pentosaceus* E3 to identify gene clusters associated with EPS biosynthesis. Additionally, the production and characterization of its novel EPS and the evaluation of the cytotoxicity, antidiabetic, anti-colon cancer, and anti-inflammatory potentials of E3-EPS. Moreover, it determines the relation between the EPS biosynthetic genotype and phenotype.

## Materials and methods

### Bacterial strain and culture conditions

The marine probiotic isolate identified as *Pediococcus pentosaceus* E3 (NCBI Accession no. JAVLVE000000000) was isolated from the gut of marine shrimp samples collected from the Mediterranean Sea. *P. pentosaceus* E3 whole genome sequence was analyzed [47]. It was activated at 37 °C for 24 h in De Man Rogosa and Sharpe broth medium (MRS; Merck, Germany).

### E3-EPS biosynthesis gene cluster identification

The EPS biosynthesis gene cluster in the marine strain *P. pentosaceus* E3 was identified using the egg-NOG Mapper tool (Version 5.0) (http://eggnog-mapper.embl.de). Protein sequence homologies were analyzed using the protein Basic Local Alignment Search Tool (BLASTp). The Nucleotide sugar biosynthesis pathway was predicted using the Kyoto Encyclopedia of Genes and Genomes (KEGG; https://www.kegg.jp).

### Exopolysaccharide (EPS) production

For EPS production, the marine isolate *P. pentosaceus* E3 was cultured in 1 L MRS broth containing 1.0% sucrose for 48 h at 37 °C [12]. After incubation, the bacterial cells were separated by centrifugation at 4000 rpm for 20 min at 4z °C. The supernatant was treated with 10% (w/v) trichloroacetic acid (TCA) for 30 min for protein degradation. After that, it was centrifuged at 3500 rpm for 20 min at 4 °C to remove proteins. Three times the volume of ice-cold absolute ethanol was added to the protein-free supernatant and left overnight at 4 °C for EPS precipitation. Following centrifugation, the pellets were collected, dissolved in deionized water, and then subjected to dialysis (10–12 kDa, Sigma-Aldrich, USA) for 48 h at 4 °C, and the water was changed twice [48, 49].

### Physicochemical characterization

#### Total carbohydrate content

The total carbohydrate content of E3-EPS was determined following dialysis using the phenol sulfuric acid assay [11]. Briefly, 0.5 mL of phenol (2.5% w/v) was added to 0.5 mL of E3-EPS (5 mg/mL), followed by the addition of 2.5 mL of concentrated sulfuric acid. The reaction mixture was incubated at room temperature for 30 min; after that, the absorbance was measured at 490 nm using a spectrophotometer (Jenway 6305, UK). A standard plot was generated using glucose concentrations (10–100 µg/mL).

#### Fourier Transform Infrared Spectroscopy (FTIR)

The dried E3-EPS was investigated with an FTIR spectrophotometer (Bruker Tensor 27, Germany) to identify the main functional groups. The E3-EPS powder (10 mg) was mixed with potassium bromide (KBr) to form a pellet, and then it was loaded onto the single-crystal germanium of the FTIR spectrometer. The FTIR spectra were obtained within the frequency range of 4000—400 cm^−1^ with a resolution of 4.0 cm^−1^ and 64 scans [20].

#### Scanning Electron Microscopy (SEM) and Energy Dispersive X-ray Spectroscopy (EDX)

E3-EPS surface morphology and EDX elemental analysis were examined with a scanning electron microscope spectrometer (SEM; JSM-IT 200, JEOL, Japan). Before imaging, the dried E3-EPS biopolymer (10 mg) was coated with a layer of gold (15 Å) for 2 min at an accelerating voltage of 20.0 kV using physical vapor deposition, then it was subjected to SEM visualization at 2000x, 5000x, and 10,000 × magnifications. The elemental composition analysis of E3-EPS was subsequently conducted, using a scanning electron microscope-energy dispersive X-ray (SEM–EDX) spectrometer. The emitted X-rays were used to determine the atomic composition and weight of the detected elements [46].

#### High-Performance Liquid Chromatography (HPLC)

The monosaccharide composition of the E3-EPS was determined after hydrolysis through HPLC analysis. A 10 mg of E3-EPS was treated with 2 mL of 2 M trifluoroacetic acid (TFA) in a sealed glass tube and heated at 100 °C for 6 h. The hydrolysate was evaporated, dissolved in deionized water, and filtered through a 0.22 μm filter. Chromatographic analysis was performed using high-performance liquid chromatography (HPLC; Shimadzu 1100, Singapore). The EPS hydrolysate was injected into a reversed-phase C18 column (50 mm × 3 mm, 2.7 μm, Agilent, Poroshell 120EC). The UV detection wavelength was 355 nm. The mobile phase, composed of solvent A, contained phosphoric acid (0.5%), 1-butylamine (0.2%), and tetrahydrofuran (1.0%) mixed in water, while solvent B contained acetonitrile (50%) and solvent A (50%) with a 1 mL/min flow rate and the temperature was maintained at 25 °C. The elution program consisted of a 5% B isocratic phase for 25 min, followed by a linear increase to 15% B at 50 min. The column was washed for 15 min with 100% B and equilibrated for 15 min at the initial conditions to guarantee reproducibility across runs [2].

#### Structural Studies Using Nuclear Magnetic Resonance Spectroscopy (NMR)

The E3-EPS sample (20 mg) was dissolved in 1 mL of DMSO-d₆ and analyzed using a Bruker Avance III 400 MHz NMR spectrometer (Germany) at 25 °C. 1D ^1^H and ^13^C NMR spectra were recorded to identify the proton and carbon environments. All spectra were processed using TopSpin (Version 3.6), and peak assignments were made based on chemical shift data and literature comparison for common saccharides.

#### Thermogravimetric (TGA) and Differential Scanning Calorimetric (DSC) analyses

Thermogravimetric and differential colorimetric analyses were performed using the TGA and DSC (V20.9; SDT Q600, USA). To determine weight loss, 14 mg of dried E3-EPS was subjected to a platinum crucible at temperatures ranging from 50 to 500 °C at a heating rate of 10 °C/min under a nitrogen atmosphere. The relation between temperature and weight loss was recorded to investigate the response of the E3-EPS biopolymer to heating.

### Biological activities of E3-EPS

#### Cytotoxic activity

The cytotoxic impact of E3-EPS was evaluated using a 3-(4,5-dimethylthiazole-2-yl)−2,5-diphenyltetrazolium bromide (MTT) assay against the Caco-2 colon cancer cell line (ATCC HTB-37) responsible for colorectal adenocarcinoma. A 96-well tissue culture plate was inoculated with 1 X 10^5^ cells/mL (100 μL/well) and incubated at 37 °C for 24 h. The formed cell monolayer was washed twice with the wash media, and 0.1 mL of two-fold dilutions of E3-EPS, prepared in RPMI medium with 2.0% serum (maintenance medium), was added to each well and then incubated at 37 °C. A 20 μL of MTT solution (Bio Basic, Canada) was added to each well, shaken at 150 rpm for 5 min, and incubated at 37 °C for 4 h in the presence of 5% CO_2_. The plate was dried to remove media, and the MTT metabolic product was resuspended in 200 μL DMSO and shaken at 150 rpm for 5 min. Finally, the optical density was recorded at a wavelength of 560 nm.

#### Antidiabetic activity

The α-amylase inhibitory activity of different concentrations of the E3-EPS was performed using the α-amylase Inhibitor Screening Kit (BioVision, USA) according to the manufacturer’s protocol, and acarbose was used as a reference. The absorbance was measured at 405 nm, and the inhibition percentage was calculated according to the following equation:

$$\text{Inhibition }({\%})=((\text{A}-\text{B})/\text{A})\text{ X }100$$

where A is the absorbance of the enzyme control, and B is the absorbance of the sample.

#### Anti-inflammatory activity

The anti-inflammatory properties of E3-EPS were in vitro assessed against lipopolysaccharide (LPS)-induced RAW264.7 macrophage cells, which stimulate the release of inflammatory cytokines, including TNF-α and IL-6. The total RNA was extracted from the cells treated with E3-EPS using an RNA extraction kit (Qiagen, USA) and reverse-transcribed to generate cDNA. The expression of cytokine genes was determined using the Script One-Step RT-PCR Kit (Bio-Rad, USA) according to the manufacturer’s protocol using a real-time PCR cycler (Rotor-Gene, India). The primer sequences used are listed in Table 1. The gene expression analysis data were quantified as fold change and compared to a calibrator (control) after normalization using the housekeeping gene, β-actin. The quantity of target genes was determined using the comparative threshold cycle (Ct) approach.

**Table 1:** Oligonucleotide primer sequences were used in this study

Gene	Primers	Reference
*TNFα*	F 5’-CTCTTCTGCCTGCTGCACTTTG-3’ R 5'-ATGGGCTACAGGCTTGTCACTC-3'	(Zhang et al. 2023) [53]
*IL6*	F 5’-GCTCTACACCTCCAATGTGACC-3’ R 5'-CTGCCGAGATTTGAGCCTCATG-3'	(Peng et al. 2022) [31]
*ACTβ*	F 5’-GCACCACACCTTCTACAATG-3’ R 5’-TGCTTGCTGATCCACATCTG-3’	(Fawzy 2019) [14]

### Statistical analysis

The trials were performed three times, and the results were reported as the mean ± standard deviation (SD). The data was processed utilizing Microsoft Excel 2010, and statistical significance was determined by one-way variance analysis (ANOVA). The observed differences were statistically significant at a significance level of *p* < 0.05.

## Results and discussion

### Prediction of EPS Biosynthesis-Related Genes Based on Genome Analysis

**Fig. 1:**
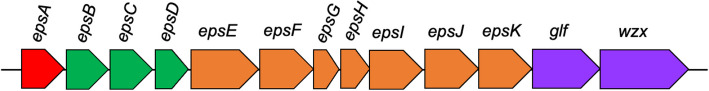
Schematic representation of the gene cluster involved in E3-EPS biosynthesis in marine *Pediococcus pentosaceus* E3

Limited research has been undertaken on the EPS of probiotic bacteria; nevertheless, new findings indicate that they are significant substances due to their health-promoting properties and economic utility across several food and pharmaceutical sectors [9]. *P. pentosaceus* E3 is derived from a marine habitat and has been recognized for its probiotic properties. The whole genome sequence examination of the marine-safe probiotic *P. pentosaceus* E3 revealed the presence of chromosomally located *eps* gene clusters encoding the Wzx/Wzy-dependent biosynthetic pathway. This pathway allows the production of heteropolysaccharides containing two or more different monosaccharide units [50]. Previously, a study reported the occurrence of *eps* gene clusters in *P. pentosaceus* LP28 and *P. pentosaceus* LL-07 [27, 44]. The *eps* gene clusters in *P. pentosaceus* E3 contain 13 genes organized into four parts, including the transcriptional regulatory region of EPS expression (*epsA*), the chain length determination region (*epsBCD*), genes encoding glycosyl transferase (GTF) that catalyze the biosynthesis of repeated units and polymerization of the EPS (*epsEFGHIJK*), and gene encoding export/flippase that occur within the EPS operon and is employed in the transportation of the EPS outside the cell membrane (*wzx*) (Table 2, Fig. 1). A previous study reported the presence of the conserved *epsBCD* gene cluster in *P. pentosaceus* SL4 [10], similar to that in *Lactococcus lactis* B40. Moreover, the CpsD/CapB family tyrosine-protein kinase has a significant regulatory role and acts as a domain protein involved in EPS biosynthesis [16]. Additionally, GTFs catalyze the transfer of activated monosaccharides to carbohydrates during the biosynthesis of EPS [5]. Moreover, the Wzy protein catalyzes polymerization in the periplasmic region [3, 40]. Once synthesized, the EPS is transported across the cell membrane and secreted into the extracellular environment by the action of the Wzx protein (flippase) [29].

**Table 2:** Exopolysaccharide (EPS) biosynthesis gene cluster of the marine isolate *P. pentosaceus* E3 using BLASTp search

Protein	Length (aa)	Gene	Function	Identity (%)	Query Coverage (%)	Accession number
Wzz/FepE/Etk N-terminal domain-containing protein	261	*epsA*	Capsular polysaccharide biosynthesis protein	99.62	100	WP_146419359
CpsD/CapB family tyrosine-protein kinase	243	*epsB*	Tyrosine-protein kinase	100	100	WP_146419358
CpsB/CapC family capsule biosynthesis tyrosine phosphatase	262	*epsC*	Capsule biosynthesis tyrosine phosphate	99.24	99	WP_146419688
Bacterial sugar transferase	221	*epsD*	Sugar transferase	99.54	100	WP_146419357
Polysaccharide polymerase	386	*epsE*	Polysaccharide polymerase	30.41	97	WP_220689059
Glycosyltransferase family GT2	301	*epsF*	Glycosyltransferase	100	100	WP_146419355
PssD/Cps14F family polysaccharide biosynthesis glycosyltransferase	150	*epsG*	Polysaccharide biosynthesis glycosyltransferase	99.33	100	WP_146419354
UDP-N-acetylglucosamine transferase	163	*epsH*	Glycosyltransferase	100	100	WP_146419353
CDP-glycerol glycerophosphotransferase family protein	374	*epsI*	Glycerol glycerophosphotransferase	100	100	WP_146419352
Glycosyltransferase family 2 protein	330	*epsJ*	Glycosyltransferase	100	100	WP_146419351
Lipopolysaccharide 1,6-glycosyltransferase	350	*epsK*	Glycosyltransferase	99.71	100	WP_146419350
UDP-galactopyranose mutase	373	*glf*	UDP-galactopyranose mutase	99.20	100	WP_201627007
Flippase Wzx	471	*wzx*	Flippase	99.36	100	WP_146419348

### Biosynthesis of Nucleotide Sugars of *P. pentosaceus* E3

In this study, a total of 16 key enzymes involved in the nucleotide sugar biosynthesis pathway were predicted according to the KEGG metabolic pathways (Table 3). LAB consumes glucose as substrate, and glucokinase (*glk*) catalyzes the phosphorylation of the substrate to glucose-6-phosphate, which serves as a central metabolite in the glycolysis pathway [30]. The phosphorylated sugars are converted into nucleotide sugars, which are important precursors for exopolysaccharide synthesis [54]. For instance, the phosphoglucomutase enzyme, encoded by the *pgm* gene, functions in converting glucose-6-phosphate into glucose-1-phosphate in the glycolysis pathway [15]. Glucose-1-phosphate uridylyltransferase (*galU*) catalyzes the formation of UDP-glucose (an essential precursor for EPS synthesis) from glucose-1-phosphate. Moreover, UDP-glucose 4-epimerase (*galE*) and galactokinase (*galK*) catalyze the reversible conversion between UDP-glucose and UDP-galactose [54].

**Table 3:** Nucleotide sugar biosynthesis key enzymes of *P. pentosaceus* E3 are annotated in the KEGG database

Gene	Encoded protein
*glk*	Glucokinase
*glmS*	Glutamine-fructose-6-phosphate transaminase
*glmM*	Phosphoglucosamine mutase
*glmU*	Glucosamine-1-phosphate N-acetyltransferase
*glf*	UDP-galactopyranose mutase
*pgm*	Phosphoglucomutase
*pgi*	Glucose-6-phosphate isomerase
*manA*	Mannose-6-phosphate isomerase
*wecB*	UDP-N-acetylglucosamine 2-epimerase
*galE*	UDP-glucose 4-epimerase
*galT*	Udpglucose-hexose-1-phosphate uridylyltransferase
*galU, galF*	UTP-glucose-1-phosphate uridylyltransferase
*scrK*	Fructokinase
*galK*	Galactokinase
*murA*	UDP-N-acetylglucosamine 1-carboxyvinyltransferase
*murB*	UDP-N-acetylmuramate dehydrogenase

Glucose-6-phosphate isomerase (*pgi*) catalyzes the interconversion of glucose-6-phosphate and fructose-6-phosphate [22, 25]. Furthermore, UDP-N-acetylglucosamine synthesis is catalyzed by three enzymes, namely, Glutamine-fructose-6-phosphate transaminase (GlmS), Phosphoglucosamine mutase (GlmM), and Glucosamine-1-phosphate N-acetyltransferase (GlmU) [36]. Furthermore, mannose-6-phosphate isomerase is a critical enzyme that is involved in the reversible conversion of mannose-6-phosphate and fructose-6-phosphate through the production of mannose-containing polysaccharides [42]. Thus, E3-EPS is composed mainly of repeating units of glucose, galactose, and mannose. This is in accord with Ayyash et al. [4], who indicated that the EPS-M41 produced by *P. pentosaceus* M41 is composed mainly of glucose, galactose, mannose, and arabinose.

### Characterization of E3-EPS

Unlike *Lactiplantibacillus plantarum*, only a few studies have been conducted on the characterization of EPS produced by *P. pentosaceus*. To the best of our knowledge, this is the first study to produce EPS from marine *P. pentosaceus* and evaluate its biological activities. *P. pentosaceus* E3 successfully produced 400 mg/L of EPS upon being grown for 48 h at 37 °C in MRS broth medium supplemented with 1.0% sucrose. The E3-EPS yield obtained in this study is much higher than the EPS yield obtained by the isolate *P. pentosaceus* SSC–12, which produced 276.6 mg/L [13]. The marine environment provides a viable setting for novel LAB strains as they thrive in a harsh and challenging habitat subjected to extreme conditions such as high salinity, fluctuating temperature, and lack of nutrients. In response, the marine LAB tend to produce structurally unique EPS adapted to function under such harsh conditions [49]. These polymers aid in cell defense, biofilm formation, and integrity preservation in challenging environments [38]. Since EPS synthesis usually increases in response to nutritional or physical stress, the marine *P. pentosaceus* E3 is a promising source for novel EPS with potentially improved stability and bioactivity.

#### Total carbohydrate contents

The phenol–sulfuric acid technique was employed to quantify the total carbohydrate content in the produced EPS. The carbohydrate percentage was determined by UV–VIS measurement, which revealed a significant carbohydrate content of approximately 93.2% using glucose as the reference standard. The high carbohydrate contents indicate the purity of E3-EPS. The carbohydrate content acquired in this investigation significantly exceeds that reported by Fan et al. [13], where the total sugar content of the isolate *P. pentosaceus* SSC–12 was determined as 73.6%.

#### FTIR spectral analysis of the E3-EPS

**Fig. 2:**
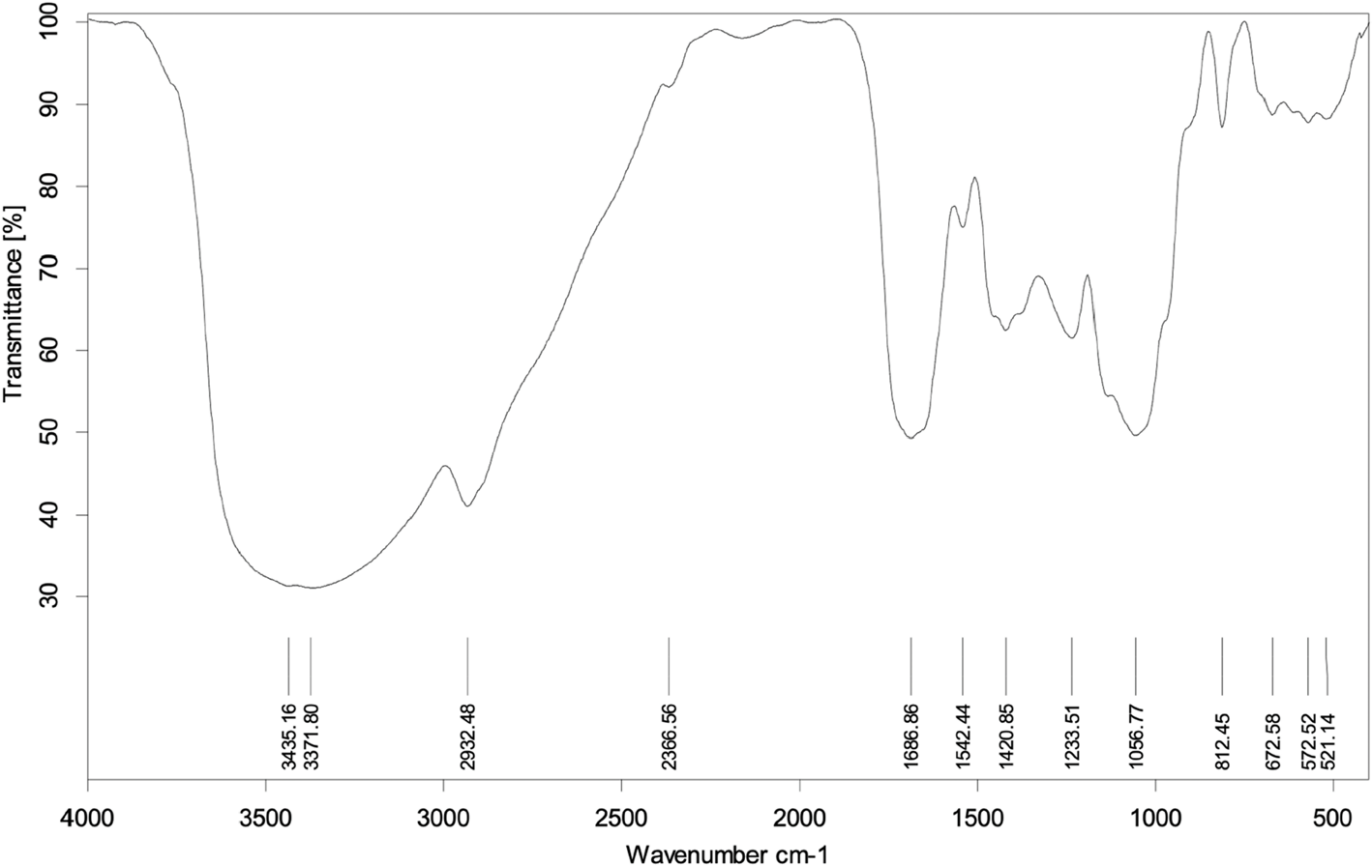
FTIR analysis of E3-EPS produced by *Pediococcus pentosaceus* E3

The specific peaks in the FTIR spectrum were used to identify the functional groups present in E3-EPS (Fig. 2). The broad peak at 3371 cm^−1^ corresponds to the presence of -OH stretching vibrations [21]. The presence of a peak at around 2932 cm^−1^ revealed the presence of C-H stretching vibrations characteristic of polysaccharides [4]. The absorption at 1686 cm^−1^ corresponds to the carbonyl group (C = O) stretching vibrations. In addition, the peak at 1420 cm^−1^ is attributed to the bending vibrations of C-H bonds. The 1233.51 cm^−1^ indicates that E3-EPS contains a pyranose ring [4]. Moreover, the absorption peak at 1056 cm^−1^ corresponds to the C–O–C stretch. Finally, the peak at 672–812 cm^−1^ is associated with the stretching vibrations of α- and β-glycosidic linkages between sugar moieties. The IR spectrum's signature in this study aligns with prior reports regarding exopolysaccharides [46, 48].

#### Morphological studies by SEM and elemental analysis using EDX spectroscopy

**Fig. 3:**
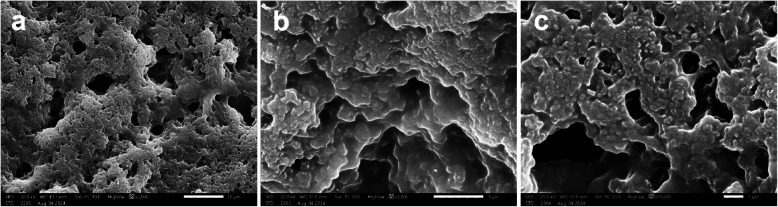
Scanning electron micrograph of E3-EPS produced by *Pediococcus pentosaceus* E3 at (**a**) 2,000 x, (**b**) 5,000 x, and (**c**) 10,000 × magnification

**Fig. 4:**
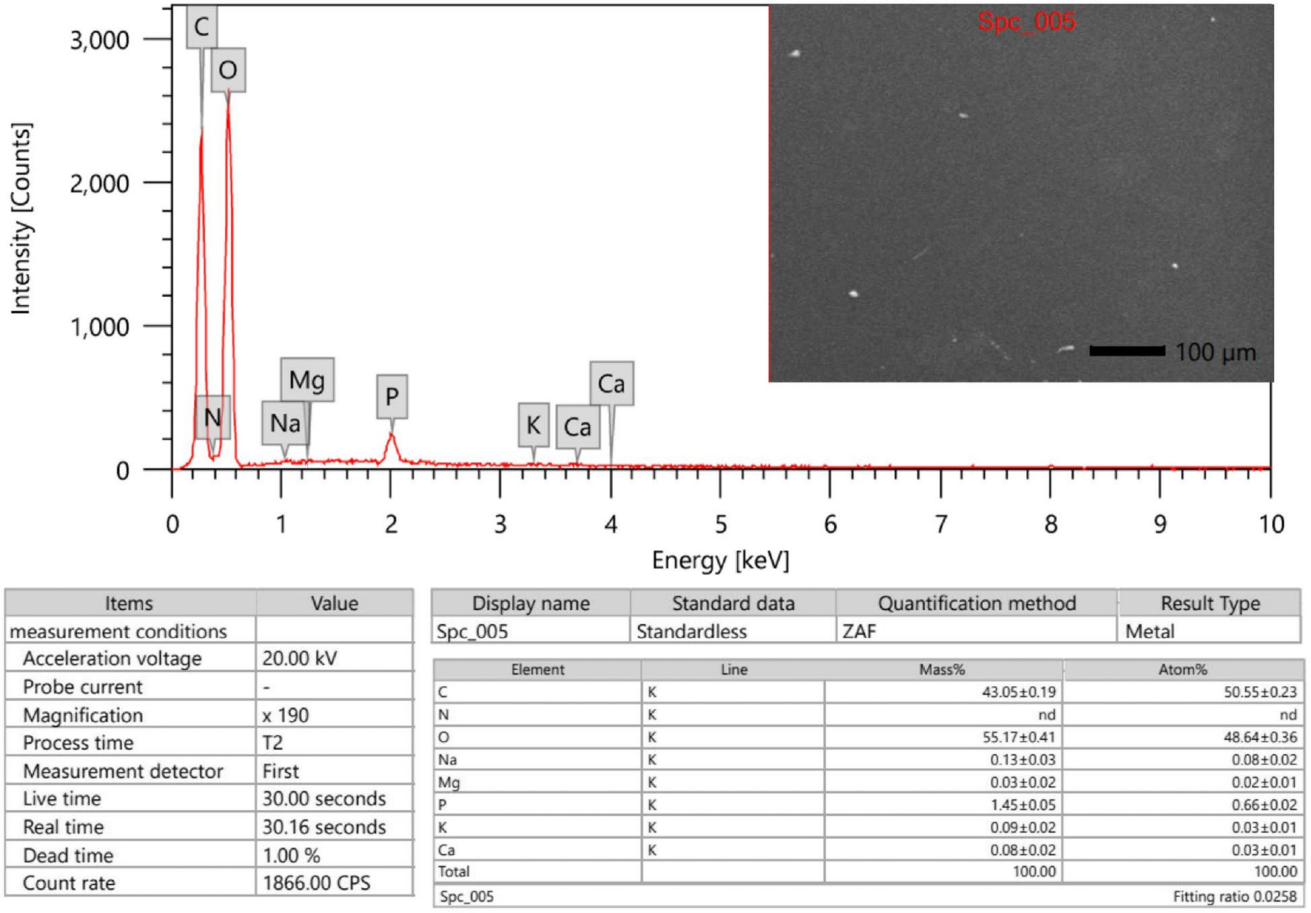
SEM–EDX analysis of E3-EPS produced by *Pediococcus pentosaceus* E3

The E3-EPS underwent structural characterization using SEM and EDX. The SEM micrographs of the E3-EPS biopolymer produced by *P. pentosaceus* E3 revealed an irregular porous morphology and an amorphous texture (Fig. 3). EDX was employed to assess the elements present in terms of weight percentage. The qualitative elemental analysis by EDX revealed the predominance of oxygen and carbon with mass ratios of 55.17 ± 0.41 and 43.05 ± 0.19 (w/w%), respectively, reflecting the high carbohydrate contents of the EPS (Fig. 4). The mass ratio of phosphorus was 1.45 ± 0.05 (w/w%), while nitrogen was not determined in the sample, indicating the purity of the EPS. Moreover, the analysis demonstrated traces of other elements, including sodium, magnesium, calcium, and potassium. These elements might be involved in how monosaccharide hydroxyl and carboxyl groups interact with one another [33].

#### Determination of monosaccharide composition by HPLC

**Fig. 5:**
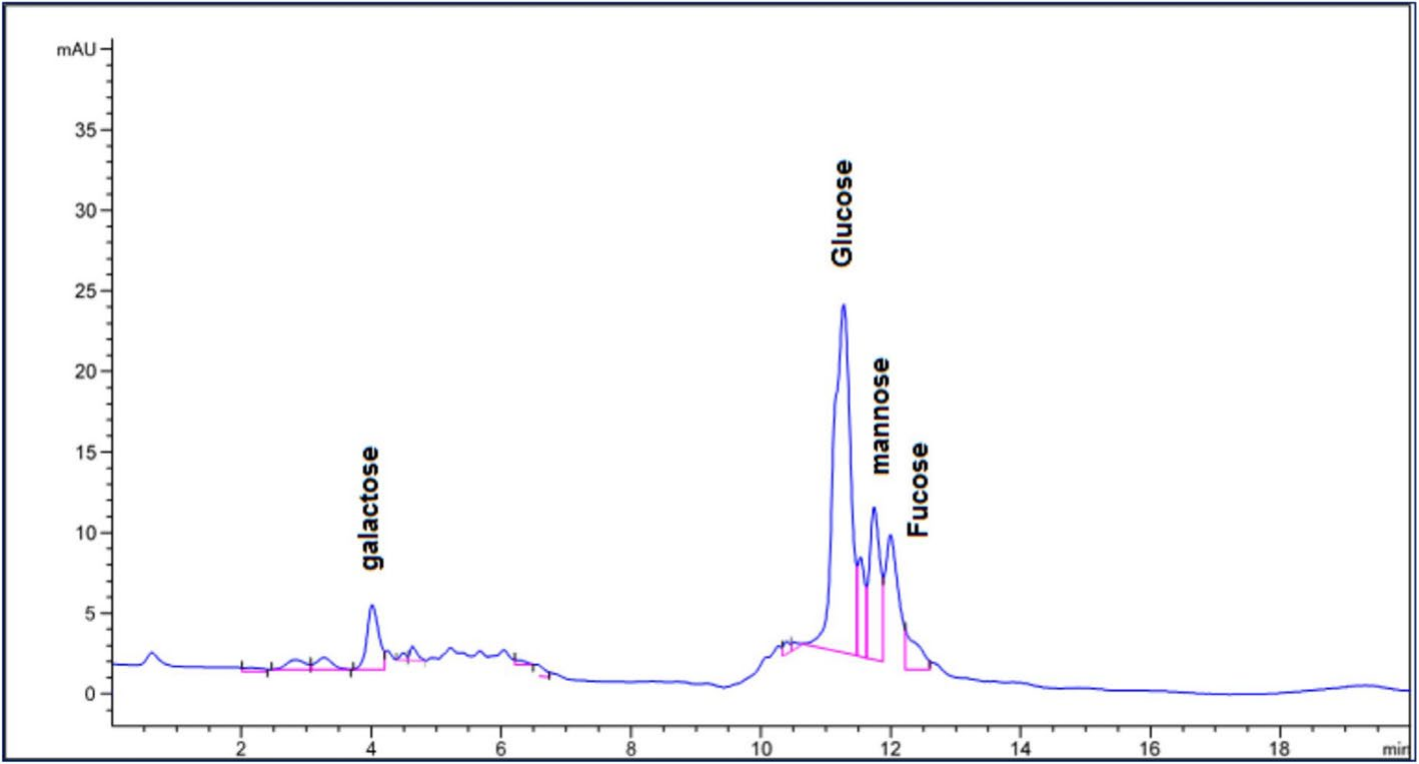
HPLC chromatogram of monosaccharide composition of E3-EPS

HPLC analysis revealed that E3-EPS was a heteropolysaccharide that contained four monosaccharides, including galactose, glucose, mannose, and fucose, in the mass percentages 27.17, 39.92, 12.10, and 9.15%, respectively. Monosaccharide peaks estimated by the HPLC method were observed in the chromatogram (Fig. 5). The results suggest that glucose and galactose were the predominant monosaccharides in the E3-EPS sample. Interestingly, fucose was detected in E3-EPS biopolymer, a rare sugar that may provide EPS with extra biological properties such as anti-inflammatory, anticancer, antioxidant, and wound healing activity [35]. Unlike the obtained results, the monosaccharide composition *of P. pentosaceus* M41 was determined by Ayyash et al. [4] as arabinose, mannose, glucose, and galactose [4]. A further study reported that EPS produced by *P. pentosaceus* LP28 acidic hydrolysate contained galactose, glucose, glucosamine, and mannose [44]. Moreover, *P. pentosaceus* SSC–12 EPS contained glucose (42.6%), mannose (28.9%), galactose (16.2%), arabinose (9.4%), and rhamnose (2.9%) [13]. The differences in monosaccharide composition and ratio within the same species are due to various factors, including culture medium and conditions [21]. Consequently, the E3-EPS produced by the marine *P. pentosaceus* E3 is a unique EPS with unique bioactivities.

#### ^1^H and ^13^C NMR spectroscopy analyses

**Fig. 6:**
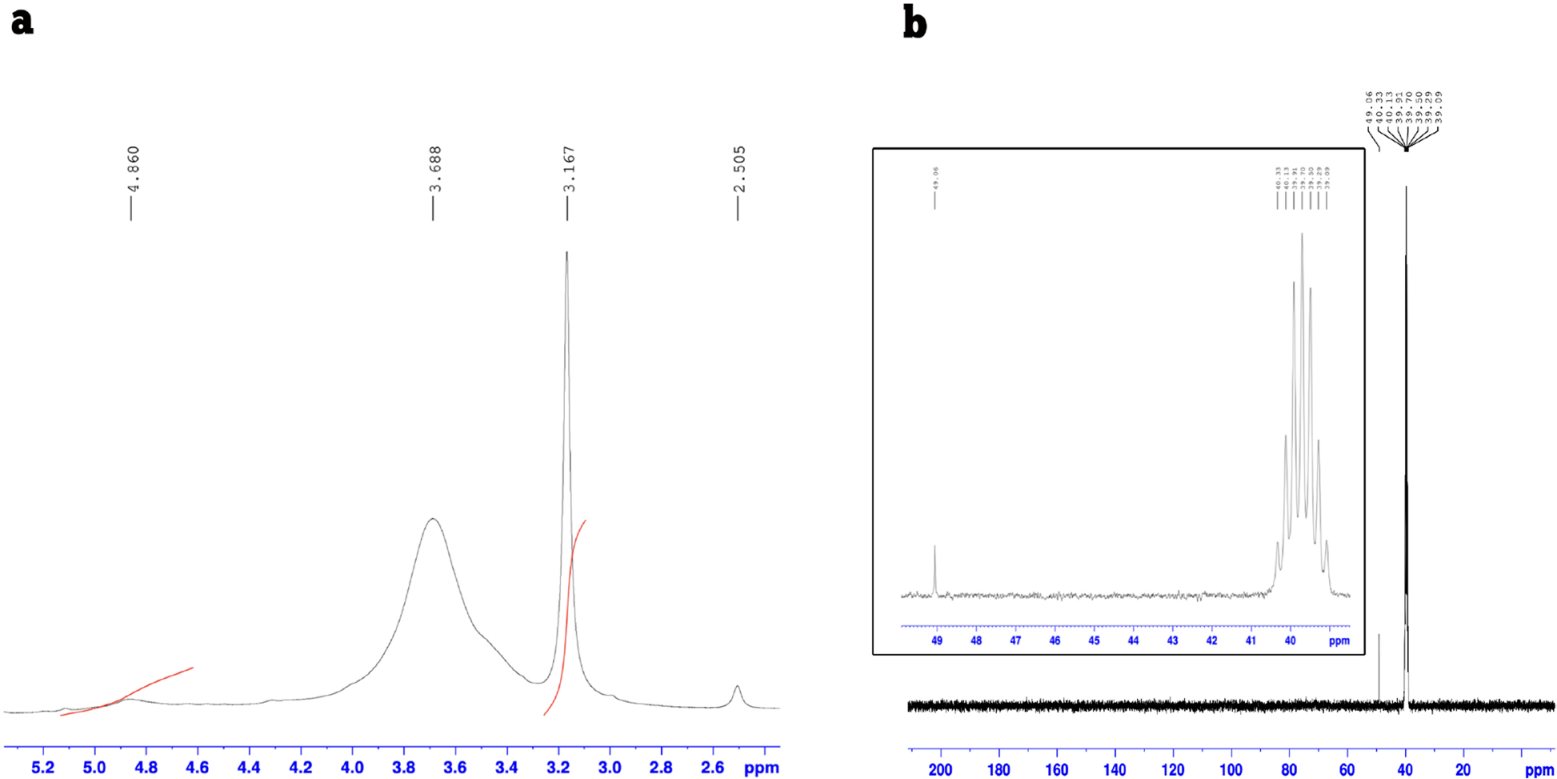
1D NMR spectra of E3-EPS by *Pediococcus pentosaceus* E3 (20 mg/mL in DMSO-d_6_ at 25 °C). (**a**) ^1^H NMR and (**b**) ^13^C NMR spectra

The structural characterization of E3-EPS using ^1^H NMR spectroscopy revealed signals in the range of δ 3.0–4.0 ppm, characteristic of the protons of hexose and pentose sugar units (Fig. 6a). A significant signal was observed at δ 4.860 ppm corresponding to the anomeric proton, characteristic of α-linked hexoses, and indicating the presence of glycosidic linkages of sugar units. An additional signal at δ 3.1 and δ 3.9 ppm is assigned to ring protons of hexopyranose residues. The ^13^C NMR spectrum (Fig. 6b) supported this with ring carbon signals in the δ 39–49 ppm range. The lack of well-resolved signals above 100 ppm could be attributed to overlapping or line-broadening effects common in polysaccharide analysis. These patterns are consistent with spectra reported for α-glucans in EPS-E8 [21] and EPS-4412 [8]. These observed chemical shift patterns are characteristic of glycosidic linkages and further confirm that the polymer is a heteropolysaccharide with an α-configuration [1].

The EPS produced by *P. pentosaceus* E3 is a branched heteropolysaccharide, which is mainly made up of glucose residues linked by α-(1 → 6) and side chains connected by α-(1 → 3) that contain galactose, mannose, and fucose. Integrated FTIR, HPLC, ^1^H and ^13^C NMR spectroscopy, and comparative genomic analysis strongly support the E3-EPS structure. Similar sugar profiles have been reported previously for EPS from *P. pentosaceus* E8 (glucose, mannose, galactose) [21] and M41 (glucose, mannose, galactose, arabinose) [4], showing a branched heteropolysaccharide composed primarily of α-(1 → 6)-linked glucose units with galactose, mannose as branching residues [4, 21].

The comprehensive analysis of the *P. pentosaceus* E3 genome revealed the presence of sixteen key genes associated with the nucleotide sugar biosynthesis pathway, which strongly supports the produced E3-EPS chemical characterization. The detected genes *glk*, *pgm*, and *galU* are responsible for converting glucose to UDP-glucose, the primary precursor for the glucan backbone formation [15, 30]. The genes *galE* and *galK* enable the reversible interconversion between UDP-glucose and UDP-galactose, which permits galactose to be incorporated into the EPS side chains [54]. Likewise, *manA* facilitates mannose-derived branching by allowing the routing of fructose-6-phosphate to mannose-6-phosphate [42]. The EPS chemical structure revealed from NMR and HPLC confirmed the dominant presence of glucose, along with galactose, mannose, and trace fucose. This composition is entirely in line with the anticipated enzymatic capabilities. Thus, the combination of genomic and chemical characterization data provides a consistent and coherent model for a branched heteropolysaccharide structure, confirming that *P. pentosaceus* E3 has functional EPS production machinery.

#### Thermostability of E3-EPS

**Fig. 7:**
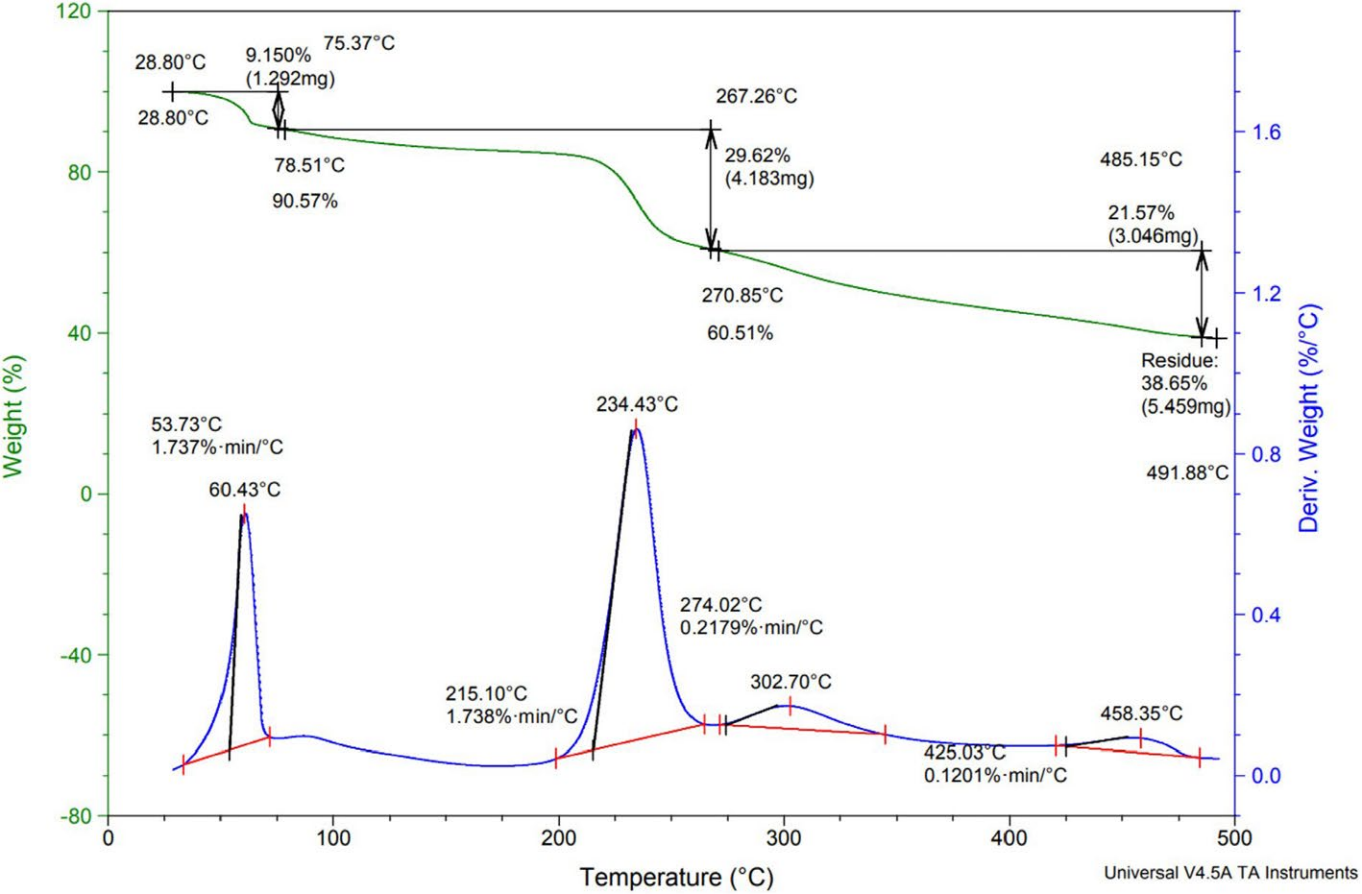
DSC-TGA analysis of E3-EPS produced by *Pediococcus pentosaceus* E3

The evaluation of thermal properties plays a significant role in understanding the physicochemical characteristics of EPS. As illustrated in Fig. 7, the E3-EPS biopolymer degrades in a stepwise manner. The initial step revealed that the weight loss at temperatures between 75.37 and 267.26 °C was about 9.15% and 29.62%, respectively. The initial loss is owing to the loss of surface-bond water molecules and the fast decomposition of the EPS [21, 45]. In the second step, weight loss was about 21.57% at 485.15 °C. This was caused by high temperature, which led to the breakage of carbon chains in the ring unit and hydrogen bonds in the EPS [21]. The remaining weight of the E3-EPS at 500 °C was about 38.65% (5.45 mg) of the original weight. The complexity of the E3-EPS molecular structure and its capacity to withstand higher degradation temperatures indicated the thermostability of the EPS produced by *P. pentosaceus* E3 and supported its use in pharmaceutical industries.

### Biological activities

The functions of the EPS are significantly influenced by its molecular weight (Mw). High Mw, for example, increases viscosity, while low Mw is useful as a bioactive molecule. *P. pentosaceus* EPS is characterized by moderate Mw, making it suitable for diverse pharmaceutical and industrial applications [4]. Therefore, the current study investigated different biological activities of E3-EPS.

#### Assessment of anticancer activity of E3-EPS

**Fig. 8:**
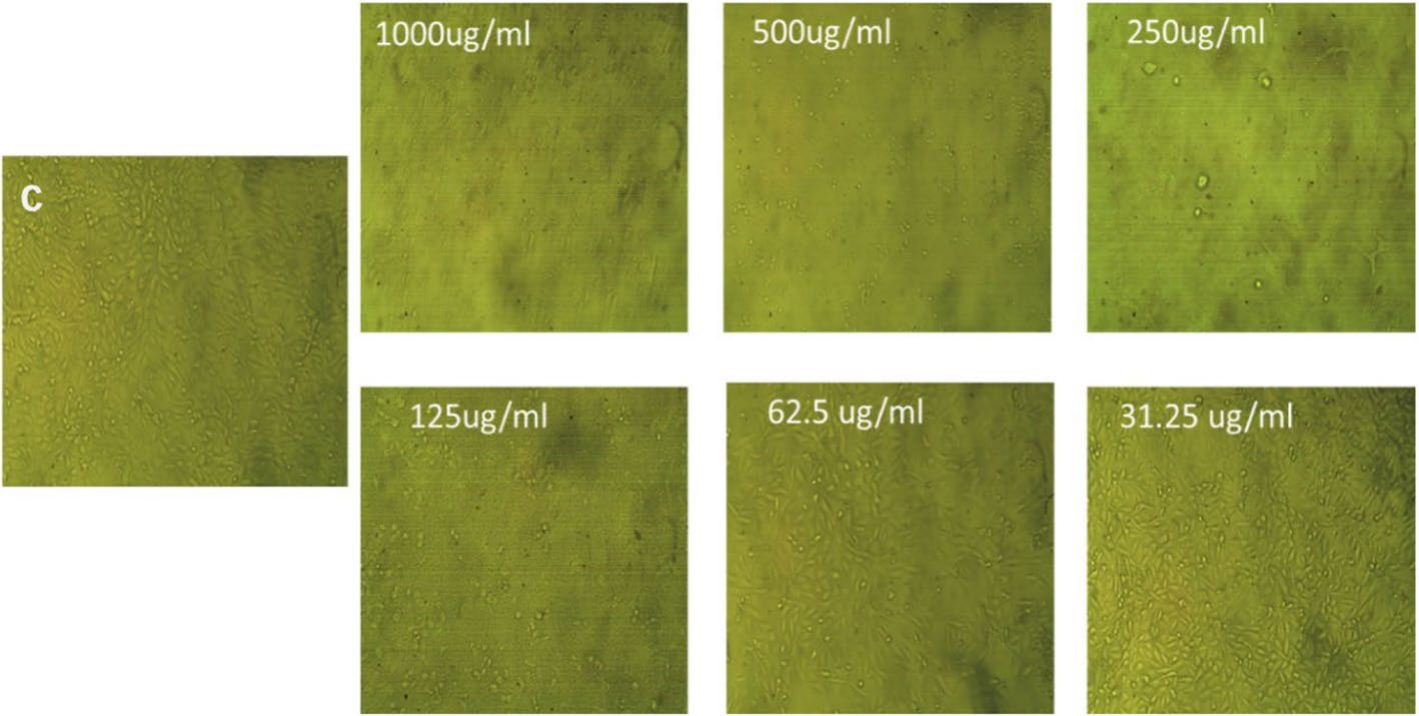
Effect of E3-EPS on colon cancer Caco-2 cells at different concentrations using the MTT assay. C is a control without E3-EPS

**Fig. 9:**
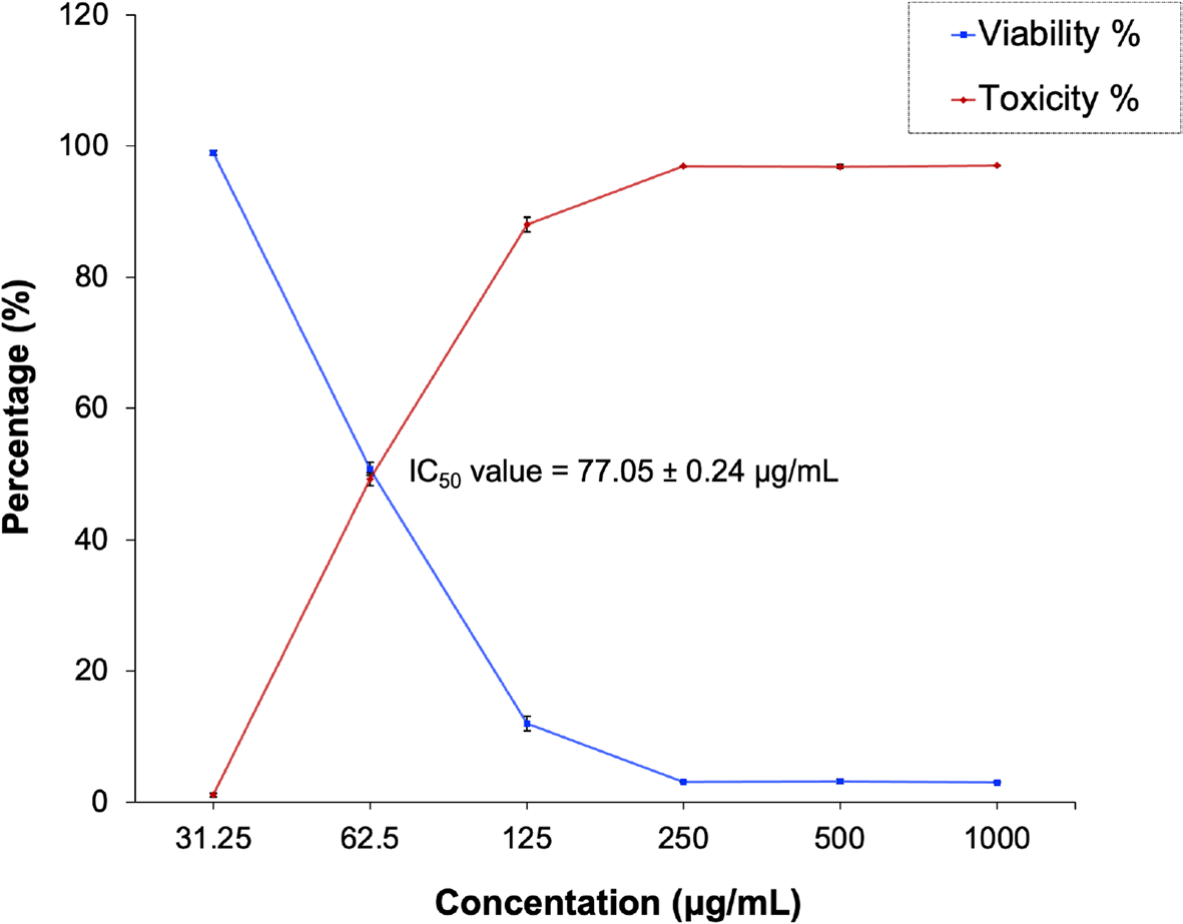
In vitro anticancer activity of E3-EPS against colon cancer cell lines using the MTT assay

The MTT assay was used to assess the anticancer efficacy of E3-EPS against colon cancer cell lines in vitro. The E3-EPS revealed a cytotoxic effect on the investigated cell lines, with the value of IC_50_ 77.05 ± 0.24 µg/mL (Figs. 8 and 9). The toxicity was approximately evaluated as 87.9% at a 125 µg/mL concentration of E3-EPS. This is in accordance with the results obtained by Ayyash et al. [4], who reported inhibition percentages of 77.89 ± 1.66 and 87.37 ± 2.44 against colon cancer cell lines at concentrations of 5 and 10 mg/mL of *P. pentosaceus* EPS-M41, respectively. Moreover, the anticancer efficacy of dextran derived from *P. pentosaceus* CRAG3 on colon cancer (HT29) cells has been shown. EPS (500 μg/mL) inhibited 65.76% of HT29 cells [41]. The precise anticancer mechanism(s) of EPS remain unclear. Multiple factors may account for E3-EPS anticancer effects, including the induction of apoptotic processes in cancer cells and the competition with growth promoters (e.g., tumor necrosis factor) for cellular receptors [4]. L-fucose is unusual and has been shown to possess anticancer, antioxidant, and immunomodulatory properties [6]. The prohibitive expense of chemically synthesizing fucose-containing polysaccharides, which hampers large-scale commercial manufacturing, has led to a growing interest in naturally occurring fucose-rich EPS. An elevation in fucose content in EPS corresponded with an enhancement in the antioxidant activity of EPS. The anticancer mechanism of fucose-rich LAB-EPS resembles that of other LAB-EPS, namely, facilitating tumour cell apoptosis and cell cycle arrest. Research indicated that fucose-enriched EPS exhibited anti-tumor bioactivity in human leukemia K562 cells via influencing mitochondria-mediated pathways. Fucose-rich EPS may decrease cytokine release by activating TLR4 and Dectin-1 receptors and create morphological alterations in dendritic cells while enhancing the expression of surface characteristic markers in murine dendritic cells [53].

#### Influence of E3-EPS on α-amylase inhibitory activity

Inhibition of α-amylase activity is related to reducing hyperglycemia in diabetic patients. The IC_50_ of E3-EPS was evaluated as 3.85 ± 0.14 µg/mL, and the inhibition rate was 58.3% at a concentration of 10 µg/mL. The inhibitory effect was increased to 82.8% by raising the concentration of E3-EPS to 100 µg/mL. Similar results were obtained by Ayyash et al. [4], who reported that the EPS produced by *P. pentosaceus* M41 at a 100 μg/mL concentration showed 86.8% and 90.8% inhibition of α-amylase and α-glucosidase activities, respectively. Moreover, Sasikumar et al. [39] showed 10% and 67% inhibition of α-amylase and α-glucosidase activities at concentrations of 100 μg/mL and 300 μg/mL of EPS generated by *L. plantarum* BR2, respectively. The inhibition of α-amylase and α-glucosidase activity is a significant indirect strategy for diabetes management, as it diminishes sugar absorption from carbohydrate hydrolysis by these enzymes. Ayyash et al. [4] proposed that the mechanism of action of the EPS may be correlated to the EPS blocking both enzymes’ active sites. Moreover, Robyt [34] revealed that the glycosidic bonds may limit the activity of α-amylase and α-glucosidase enzymes.

#### Anti-inflammatory activity

**Fig. 10:**
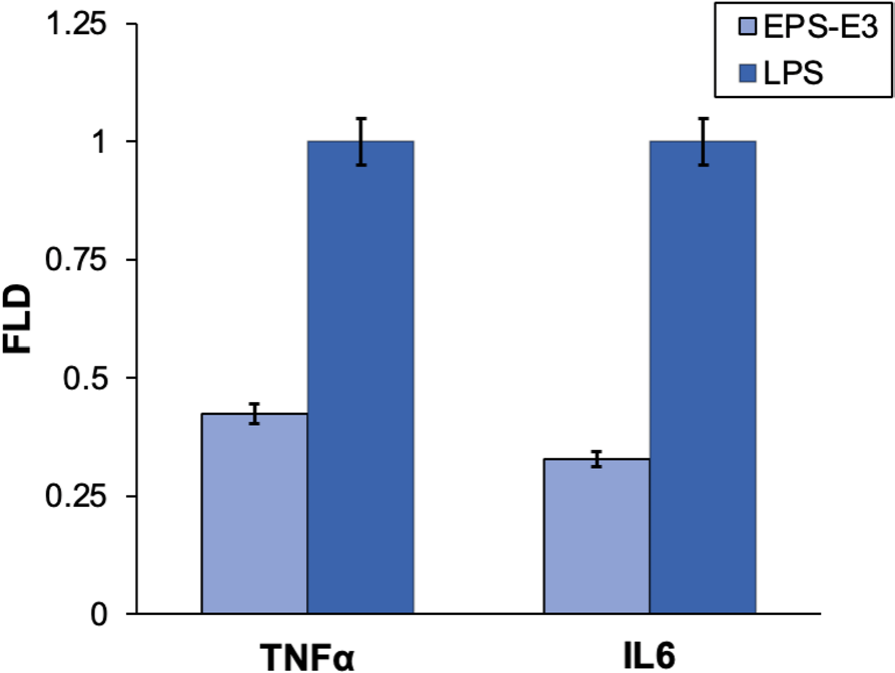
Effect of E3-EPS (20 µg/mL) on the expression of cytokines TNF-α and IL-6 after 24 h of treatment on LPS-induced RAW264.7 macrophage cells. The mRNA expression levels were measured using qRT-PCR. FLD: refers to the expression level fold change

Pro-inflammatory cytokines (TNF-α, IL-6, and IL-1β) and anti-inflammatory cytokines (IL-4, IL-10, IL-11, and IL-13) exemplify cytokines that modulate inflammatory responses through the activation or suppression of immune cells. Pro-inflammatory cytokines are particularly prevalent in immune cells, such as macrophages, monocytes, T cells, and B cells, and are crucial in the development of inflammatory disorders. In cases of inflammation, injured tissues exhibit upregulation of pro-inflammatory proteins and cytokines, with their expression levels correlating to the extent of the injury [26]. The expression levels of TNF-α and IL-6 were determined in the LPS-induced RAW264.7 macrophage cells, which stimulate the release of inflammatory cytokines. The mRNA expression levels of cytokines TNF-α and IL-6 were decreased after treatment with E3-EPS (concentration 20 µg/mL), compared to the LPS-induced level (Fig. 10). These findings indicate that E3-EPS exerts anti-inflammatory effects by decreasing pro-inflammatory mediators.

The LAB EPSs are potent bioactive agents capable of modulating the immune system. The β-D-glucose and mannose present in the EPS exhibit a triple-helix shape, resulting in higher stiffness. This distinctive shape enables interaction with immune cell receptors innovatively, demonstrating its capacity to diminish inflammation. The anti-inflammatory efficacy is considerably affected by parameters including types of sugars, molecular weight, types of glycosidic linkages, and their positions. Nonetheless, the precise mechanism by which the EPS mediates its anti-inflammatory effects remains incompletely elucidated [18].

Similar results were obtained by Lee et al. [26], who documented the anti-inflammatory properties of an EPS fraction derived from *P. pentosaceus* KFT18 in mice. These EPS successfully decreased the elevated expression of inducible nitric oxide synthase (iNOS), cyclooxygenase-2 (COX-2), and pro-inflammatory cytokines (TNF-α, IL-6, and IL-1) in the colonic tissue of mice with induced colitis. Moreover, Zhou et al. [55] declared the mechanism and structural function relation of the polysaccharides of Dendrobium officinale, which consist of mannose, glucose, galactose, and rhamnose, to reduce CRP production and downregulate blood levels of IL-6 and TNF-α by blocking the activation and proliferation of CD6 + T cells. Huang et al. [19] indicated that the EPS-LP2, which has a high concentration of glucose from *L. plantarum* DMDL 9010, suppresses the overactive immunological response via the MAPK/NF-κB pathways.

## Conclusion

The marine probiotic strain *P. pentosaceus* E3 genome studies revealed a gene cluster responsible for EPS production of 13 genes organized into four regions, including the regulatory region of EPS expression, chain length determination, EPS repeat units’ biosynthesis, and polymerization and transportation of the EPS. A total of sixteen key enzymes implicated in the nucleotide sugar biosynthesis pathway were identified through KEGG metabolic pathway analysis. E3, cultured in MRS broth with 1.0% sucrose, yielded 400 mg/L of EPS. The purified E3-EPS was found to be a heteropolysaccharide characterized by a substantial carbohydrate content (~ 93.2%), composed of five prevalent monosaccharides: galactose, glucose, mannose, and fucose linked by α-glycosidic bonds and able to withstand high temperatures. Moreover, E3-EPS showed promising bioactivities during testing against colon cancer cell lines, with an IC_50_ value of 77.05 ± 0.24 µg/mL and inhibited α-amylase activity by 58.3% and 82.8% at 10 and 100 µg/mL concentrations, respectively. E3-EPS also reduces the expression of inflammatory cytokines (TNF-α and IL-6). The findings of this study indicate that *P. pentosaceus* E3 isolated from the underexplored marine environment is a promising, safe source of a unique bioactive EPS for pharmaceutical purposes.

## Data Availability

The dataset analyzed during the current study is available in the DDBJ/ENA/GenBank database GenBank repository, with the primary accession code JAVLVE000000000.

## Abbreviations

BLAST: Basic Local Alignment Search Tool
Ct: Comparative threshold cycle
DMSO: Dimethyl sulfoxide
EDX: Energy Dispersive X-ray spectroscopy
EPS: Exopolysaccharides
FAO: Food and Agriculture Organization of the United Nations
FTIR: Fourier-Transform Infrared spectroscopy
GTF: Glycosyltransferase
HPLC: High Performance Liquid Chromatography
IBD: Inflammatory bowel disease
IL-6: Interleukin-6
LAB: Lactic Acid Bacterium
LPS: Lipopolysaccharides
MRS: De Man–Rogosa–Sharpe
MTT: 3-(4,5-Dimethylthiazole-2-yl)-2,5-diphenyltetrazolium bromide
Mw: Molecular weight
NCBI: National Center for Biotechnology Information
NMR: Nuclear Magnetic Resonance Spectroscopy
ppm: Parts per million
SEM: Scanning Electron Microscopy
TCA: Trichloroacetic acid
TFA: Trifluoroacetic acid
TNF-α: Tumor necrosis factor-alpha
WHO: World Health Organization
XRD: X-Ray Diffraction

## References

[1] Angelin J, Kavitha M. Structural characterization and in vitro anti-inflammatory activity of exopolysaccharide produced by *Pediococcus pentosaceus* 4412. Int Immunopharmacol. 2025;150:114301. 10.1016/j.intimp.2025.114301. 39970712 10.1016/j.intimp.2025.114301

[2] Anumula KR. Quantitative determination of monosaccharides in glycoproteins by high-performance liquid chromatography with highly sensitive fluorescence detection. Anal Biochem. 1994;220(2):275–83. 10.1006/abio.1994.1338. 7978269 10.1006/abio.1994.1338

[3] Ates O. Systems biology of microbial exopolysaccharides production. Front Bioeng Biotechnol. 2015;3:200. 10.3389/fbioe.2015.00200. 26734603 10.3389/fbioe.2015.00200 PMC4683990

[4] Ayyash M, Abu-Jdayil B, Olaimat A, et al. Physicochemical, bioactive and rheological properties of an exopolysaccharide produced by a probiotic *Pediococcus pentosaceus* M41. Carbohydr Polym. 2020;229:115462. 10.1016/j.carbpol.2019.115462. 31826478 10.1016/j.carbpol.2019.115462

[5] Becker A. Challenges and perspectives in combinatorial assembly of novel exopolysaccharide biosynthesis pathways. Front Microbiol. 2015;6:687. 10.3389/fmicb.2015.00687. 26217319 10.3389/fmicb.2015.00687 PMC4496566

[6] Borisova MA, Snytnikova OA, Litvinova EA, Achasova KM, Babochkina TI, Pindyurin AV, et al. Fucose ameliorates tryptophan metabolism and behavioral abnormalities in a mouse model of chronic colitis. Nutrients. 2020;12(2):445. 10.3390/nu12020445. 32053891 10.3390/nu12020445 PMC7071335

[7] Caggianiello G, Kleerebezem M, Spano G. Exopolysaccharides produced by lactic acid bacteria: from health-promoting benefits to stress tolerance mechanisms. Appl Microbiol Biotechnol. 2016;100(9):3877–86. 10.1007/s00253-016-7471-2. 27020288 10.1007/s00253-016-7471-2

[8] Chen Y, Li P, Huang W, et al. Characterization of EPS-4412 from *Pediococcus pentosaceus*. Int J Biol Macromol. 2025;240:125968. https://pubmed.ncbi.nlm.nih.gov/39970712

[9] Chung KS, Shin JS, Lee JH, et al. Protective effect of exopolysaccharide fraction from *Bacillus subtilis* against dextran sulfate sodium-induced colitis through maintenance of intestinal barrier and suppression of inflammatory responses. Int J Biol Macromol. 2021;178:363–72. 10.1016/j.ijbiomac.2021.02.186. 33652052 10.1016/j.ijbiomac.2021.02.186

[10] Dantoft SH, Bielak EM, Seo JG, Chung MJ, Jensen PR. Complete genome sequence of *Pediococcus pentosaceus* strain SL4. Genome Announc. 2013;1(6):e01106-13. 10.1128/genomeA.01106-13. 24371205 10.1128/genomeA.01106-13 PMC3873615

[11] Dubois M, Gilles K, Hamilton JK, Rebers PA, Smith F. A colorimetric method for the determination of sugars. Nature. 1951;168(4265):167. 10.1038/168167a0. 14875032 10.1038/168167a0

[12] Ermis E, Poyraz E, Dertli E, Yılmaz MT. Optimization of exopolysaccharide production of *Lactobacillus brevis* E25 using RSM and characterization. Sakarya University Journal of Science. 2020;24(1):151–60.

[13] Fan Y, Li X, Tian R, Tang R, Zhang J. Characterization and biological activity of a novel exopolysaccharide produced by *Pediococcus pentosaceus* SSC–12 from silage. Microorganisms. 2021;10(1):18. 10.3390/microorganisms10010018. 35056471 10.3390/microorganisms10010018 PMC8780647

[14] Fawzy F. Long non-coding RNA H19 as potential biomarker for HCV genotype 4 induced hepatocellular carcinoma patients. Al-Azhar J Pharm Sci. 2019;60(2):76–94. 10.21608/ajps.2019.70241.

[15] Goh YJ, Klaenhammer TR. Insights into glycogen metabolism in *Lactobacillus acidophilus*: impact on carbohydrate metabolism, stress tolerance and gut retention. Microb Cell Fact. 2014;13:94. 10.1186/s12934-014-0094-3. 25410006 10.1186/s12934-014-0094-3 PMC4243779

[16] Grangeasse C, Nessler S, Mijakovic I. Bacterial tyrosine kinases: evolution, biological function and structural insights. Philos Trans R Soc Lond B Biol Sci. 2012;367(1602):2640–55. 10.1098/rstb.2011.0424. 22889913 10.1098/rstb.2011.0424 PMC3415835

[17] Guan QA. Comprehensive review and update on the pathogenesis of inflammatory bowel disease. J immunol research. 2019;1:7247238. 10.1155/2019/7247238. 10.1155/2019/7247238 PMC6914932 31886308

[18] Hou C, Chen L, Yang L, Ji X. An insight into anti-inflammatory effects of natural polysaccharides. Int J Biol Macromol. 2020;153:248–55. 10.1016/j.ijbiomac.2020.02.315. 32114173 10.1016/j.ijbiomac.2020.02.315

[19] Huang YY, Wu JM, Wu WT, Lin JW, Liang YT, et al. Structural, antioxidant, and immunomodulatory activities of an acidic exopolysaccharide from *Lactiplantibacillus plantarum* DMDL 9010. Front Nutr. 2022;9:1073071. 10.3389/fnut.2022.1073071. 36570157 10.3389/fnut.2022.1073071 PMC9779943

[20] Ji X, Guo J, Ding D, Gao J, Hao L, Guo X, et al. Structural characterization and antioxidant activity of a novel high-molecular-weight polysaccharide from *Ziziphus jujuba* cv. muzao. J Food Meas Charact. 2022;16(3):2191–200. 10.1007/s11694-022-01288-3.

[21] Jiang G, He J, Gan L, et al. Exopolysaccharide produced by *Pediococcus pentosaceus* E8: structure, bio-activities, and its potential application. Front Microbiol. 2022;13:923522. 10.3389/fmicb.2022.923522. 35814643 10.3389/fmicb.2022.923522 PMC9257109

[22] Jiao J, Gao F, Liu J, Lv Z, Liu C. A structural basis for the functional differences between the cytosolic and plastid phosphoglucose isomerase isozymes. PLoS ONE. 2022;17(9):e0272647. 10.1371/journal.pone.0272647. 36048814 10.1371/journal.pone.0272647 PMC9436075

[23] Jurášková D, Ribeiro SC, Silva CCG. Exopolysaccharides produced by lactic acid bacteria: from biosynthesis to health-promoting properties. Foods. 2022;11(2):156. 10.3390/foods11020156. 35053888 10.3390/foods11020156 PMC8774684

[24] Lee IC, Caggianiello G, van Swam II, et al. Strain-specific features of extracellular polysaccharides and their impact on *Lactobacillus plantarum*-host interactions. Appl Environ Microbiol. 2016;82(13):3959–70. 10.1128/AEM.00306-16. 27107126 10.1128/AEM.00306-16 PMC4907176

[25] Lee JH, Chang KZ, Patel V, Jeffery CJ. Crystal structure of rabbit phosphoglucose isomerase complexed with its substrate D-fructose 6-phosphate. Biochemistry. 2001;40(26):7799–805. 10.1021/bi002916o. 11425306 10.1021/bi002916o

[26] Lee JH, Chung KS, Shin JS, Jung SH, Lee S, Lee MK, et al. Anti-colitic effect of an exopolysaccharide fraction from *Pediococcus pentosaceus* KFT-18 on dextran sulfate sodium-induced colitis through suppression of inflammatory mediators. Polymers (Basel). 2022;14(17):3594. 10.3390/polym14173594. 36080669 10.3390/polym14173594 PMC9460603

[27] Lu K, Wang X, Zhou Y, Zhu Q. Genomic characterization and probiotic potential assessment of an exopolysaccharide-producing strain *Pediococcus pentosaceus* LL-07 isolated from fermented meat. BMC Microbiol. 2024;24(1):142. 10.1186/s12866-024-03304-6. 38664612 10.1186/s12866-024-03304-6 PMC11044368

[28] Nguyen PT, Nguyen TT, Bui DC, Hong PT, Hoang QK, Nguyen HT. Exopolysaccharide production by lactic acid bacteria: the manipulation of environmental stresses for industrial applications. AIMS Microbiol. 2020;6(4):451–69. 10.3934/microbiol.2020027. 33364538 10.3934/microbiol.2020027 PMC7755584

[29] Osemwegie OO, Adetunji CO, Ayeni EA, et al. Exopolysaccharides from bacteria and fungi: current status and perspectives in Africa. Heliyon. 2020;6(6):e04205. 10.1016/j.heliyon.2020.e04205. 32577572 10.1016/j.heliyon.2020.e04205 PMC7303563

[30] Papagianni M. Metabolic engineering of lactic acid bacteria for the production of industrially important compounds. Comput Struct Biotechnol J. 2012;3:e201210003. 10.5936/csbj.201210003. 24688663 10.5936/csbj.201210003 PMC3962192

[31] Peng K, Deng N, Meng Y, et al. Alpha-Momorcharin Inhibits Proinflammatory Cytokine Expression by M1 Macrophages but Not Anti-Inflammatory Cytokine Expression by M2 Macrophages. J Inflamm Res. 2022;15:4853–72. 10.2147/JIR.S372306. 36042868 10.2147/JIR.S372306 PMC9420447

[32] Polak-Berecka M, Waśko A, Paduch R, Skrzypek T, Sroka-Bartnicka A. The effect of cell surface components on adhesion ability of *Lactobacillus rhamnosus*. Antonie Van Leeuwenhoek. 2014;106(4):751–62. 10.1007/s10482-014-0245-x. 25090959 10.1007/s10482-014-0245-x PMC4158178

[33] Prajapati D, Bhatt A, Gupte A. Evaluation of bioactive attributes and emulsification potential of exopolysaccharide produced by a brown-rot fungus *Fomitopsis meliae* AGDP-2. Appl Biochem Biotechnol. 2023;195(5):2974–92. 10.1007/s12010-022-04257-0. 36462111 10.1007/s12010-022-04257-0

[34] Robyt JF. Inhibition, activation, and stabilization of α-amylase family enzymes. Biol Bratislava. 2005;60(16):17–26.

[35] Roca C, Alves VD, Freitas F, Reis MA. Exopolysaccharides enriched in rare sugars: bacterial sources, production, and applications. Front Microbiol. 2015;6:288. 10.3389/fmicb.2015.00288. 25914689 10.3389/fmicb.2015.00288 PMC4392319

[36] Rodríguez-Díaz J, Rubio-del-Campo A, Yebra MJ. Metabolic engineering of *Lactobacillus casei* for production of UDP-N-acetylglucosamine. Biotechnol Bioeng. 2012;109(7):1704–12. 10.1002/bit.24475. 22383248 10.1002/bit.24475

[37] Rohm TV, Meier DT, Olefsky JM, Donath MY. Inflammation in obesity, diabetes, and related disorders. Immunity. 2022;55(1):31–55. 10.1016/j.immuni.2021.12.013. 35021057 10.1016/j.immuni.2021.12.013 PMC8773457

[38] Salimi F, Farrokh P. Recent advances in the biological activities of microbial exopolysaccharides. World J Microbiol Biotechnol. 2023;39(8):213. 37256348 10.1007/s11274-023-03660-x PMC10230149

[39] Sasikumar K, Kozhummal Vaikkath D, Devendra L, Nampoothiri KM. An exopolysaccharide (EPS) from a *Lactobacillus plantarum* BR2 with potential benefits for making functional foods. Bioresour Technol. 2017;241:1152–6. 10.1016/j.biortech.2017.05.075. 28579175 10.1016/j.biortech.2017.05.075

[40] Schmid J, Sieber V. Enzymatic transformations involved in the biosynthesis of microbial exo-polysaccharides based on the assembly of repeat units. Chembiochem. 2015;16(8):1141–7. 10.1002/cbic.201500035. 25873567 10.1002/cbic.201500035

[41] Shukla R, Goyal A. Novel dextran from *Pediococcus pentosaceus* CRAG3 isolated from fermented cucumber with anti-cancer properties. Int J Biol Macromol. 2013;62:352–7. 10.1016/j.ijbiomac.2013.09.043. 24095664 10.1016/j.ijbiomac.2013.09.043

[42] Sigdel S, Singh R, Kim TS, et al. Characterization of a mannose-6-phosphate isomerase from *Bacillus amyloliquefaciens* and its application in fructose-6-phosphate production. PLoS ONE. 2015;10(7):e0131585. 10.1371/journal.pone.0131585. 26171785 10.1371/journal.pone.0131585 PMC4718643

[43] Tsalamandris S, Antonopoulos AS, Oikonomou E, Papamikroulis GA, Vogiatzi G, Papaioannou S, et al. The role of inflammation in diabetes: current concepts and future perspectives. Eur Cardiol Rev. 2019;14(1):50. 10.15420/ecr.2018.33.1. 10.15420/ecr.2018.33.1 PMC6523054 31131037

[44] Yasutake T, Kumagai T, Inoue A, et al. Characterization of the LP28 strain-specific exopolysaccharide biosynthetic gene cluster found in the whole circular genome of *Pediococcus pentosaceus*. Biochem Biophys Rep. 2016;5:266–71. 10.1016/j.bbrep.2016.01.004. 28955833 10.1016/j.bbrep.2016.01.004 PMC5600453

[45] Yu L, Ye G, Qi X, et al. Purification, characterization and probiotic proliferation effect of exopolysaccharides produced by *Lactiplantibacillus plantarum* HDC-01 isolated from sauerkraut. Front Microbiol. 2023;14:1210302. 10.3389/fmicb.2023.1210302. 37440877 10.3389/fmicb.2023.1210302 PMC10333699

[46] Zaghloul EH, Abdel-Latif HH, Elsayis A, Hassan SWM. Production and characterization of novel marine black yeast’s exopolysaccharide with potential antiradical and anticancer prospects. Microb Cell Fact. 2024;23(1):60. 10.1186/s12934-024-02332-1. 38388439 10.1186/s12934-024-02332-1 PMC10882794

[47] Zaghloul EH, Halfawy NME. Marine *Pediococcus pentosaceus* E3 probiotic properties, whole-genome sequence analysis, and safety assessment. Probiotics Antimicrob Proteins. 2024. 10.1007/s12602-024-10283-7. 38748306 10.1007/s12602-024-10283-7 PMC11573859

[48] Zaghloul EH, Ibrahim MIA, Zaghloul HAH. Antibacterial activity of exopolysaccharide produced by bee gut-resident *Enterococcus* sp. BE11 against marine fish pathogens. BMC Microbiol. 2023;23(1):231. 10.1186/s12866-023-02977-9. 37612642 10.1186/s12866-023-02977-9 PMC10463787

[49] Zaghloul EH, Ibrahim MIA. Production and characterization of exopolysaccharide from newly isolated marine probiotic Lactiplantibacillus plantarum EI6 with in vitro wound healing activity. Front Microbiol. 2022;13:903363. 10.3389/fmicb.2022.903363. (2022 May 13**.**). 35668753 10.3389/fmicb.2022.903363 PMC9164304

[50] Zeidan AA, Poulsen VK, Janzen T, et al. Polysaccharide production by lactic acid bacteria: from genes to industrial applications. FEMS Microbiol Rev. 2017;41(Supp_1):S168–200. 10.1093/femsre/fux017. 28830087 10.1093/femsre/fux017

[51] Zhang Q, Wang J, Sun Q, et al. Characterization and antioxidant activity of released exopolysaccharide from potential probiotic *Leuconostoc mesenteroides* LM187. J Microbiol Biotechnol. 2021;31(8):1144–53. 10.4014/jmb.2103.03055. 34226411 10.4014/jmb.2103.03055 PMC9705892

[52] Zhang W, Zhao Y, Zhang F, et al. The use of anti-inflammatory drugs in the treatment of people with severe coronavirus disease 2019 (COVID-19): the perspectives of clinical immunologists from China. Clin Immunol. 2020;214:108393. 10.1016/j.clim.2020.108393. 32222466 10.1016/j.clim.2020.108393 PMC7102614

[53] Zhang X, Yang Z, Pan T, et al. SARS-CoV-2 Nsp8 suppresses MDA5 antiviral immune responses by impairing TRIM4-mediated K63-linked polyubiquitination. PLoS Pathog. 2023;19(11):e1011792. 10.1371/journal.ppat.1011792. 37956198 10.1371/journal.ppat.1011792 PMC10681309

[54] Zhao X, Liang Q, Song X, Zhang Y. Whole genome sequence of *Lactiplantibacillus plantarum* MC5 and comparative analysis of eps gene clusters. Front Microbiol. 2023;14:1146566. 10.3389/fmicb.2023.1146566. 37200914 10.3389/fmicb.2023.1146566 PMC10185785

[55] Zhou W, Tao W, Wang M, Liu W, Xing J, Yang Y. *Dendrobium officinale* Xianhu 2 polysaccharide helps forming a healthy gut microbiota and improving host immune system: an in vitro and in vivo study. Food Chem. 2023;401:134211. 10.1016/j.foodchem.2022.134211. 36122490 10.1016/j.foodchem.2022.134211

